# Supplementary material for: Impact of an Enhanced Transtheoretical Model Intervention (ETMI) Workshop on the Attitudes and Beliefs Regarding Low Back Pain of Primary Care Physicians in the Israeli Navy
Source: Int J Environ Res Public Health. 2023 Mar 9;20(6):4854. doi: 10.3390/ijerph20064854 (PMC10049028; doi:10.3390/ijerph20064854)
Supplement: Supplementary file 1 [file ijerph-20-04854-s001.zip › ijerph-2177639-supplementary.pdf]

**Table S1**. Primary care physicians self-reported clinical behavior for treating chronic LBP (combined cohort).

| | N (%) | Never | Rarely | Sometimes | Often | Always |
|---|---|---|---|---|---|---|
| Provide Medication Prescription | Anti-Inflammatory | 1 (3.1) | 2 (6.3) | 12 (37.5) | 15 (46.9) | 2 (6.3) |
| | Opiates | 12 (37.5) | 15 (46.9) | 5 (15.6) | | |
| | Over The Counter Pain Medication | | | 9 (28.1) | 19 (59.4) | 4 (12.5) |
| | Anti-Depressants | 14 (43.8) | 13 (40.6) | 5 (15.6) | | |
| | Benzodiazepine | | | | | |
| | Anti-Epileptic (gabapentine, lyrica) | 16 (50) | 11 (34.4) | 5 (15.6) | | |
| | Cannabis referral | 29 (90.6) | 2 (6.3) | 1 (3.1) | | |
| Send for Tests | X-Ray | 5 (15.6) | 15 (46.9) | 12 (37.5) | | |
| | CT | 3 (9.4) | 22 (68.8) | 7 (21.9) | | |
| | MRI | 23 (71.9) | 7 (21.9) | 2 (6.3) | | |
| | Bone Scan | 20 (62.5) | 9 (28.1) | 3 (9.4) | | |
| | EMG | 19 (59.4) | 11 (34.4) | 2 (6.3) | | |
| | Physiotherapy | 2 (6.3) | 1 (3.1) | 14 (43.8) | 13 (40.6) | 2 (6.3) |

| | | | | | | |
|---|---|---|---|---|---|---|
| Refer for Treatment | Alternative Treatment | 9 (28.1) | 4 (12.5) | 17 (53.1) | 2 (6.3) | |
| | Emergency Department | 7 (21.9) | 23 (71.9) | 2 (6.3) | | |
| | Pain Clinic | 17 (53.1) | 11 (34.1) | 4 (12.5) | | |
| Examination and Treatment | Manual Examination | | | | 5 (15.6) | 27 (84.4) |
| | Dry Needling | 26 (81.3) | 2 (6.3) | 1 (3.1) | 3 (9.4) | |
| | Reassure the Patient | 2 (6.3) | 4 (12.5) | 8 (25) | 14 (43.8) | 4 (12.5) |
| | Physical Activity Recommendation | 1 (3.1) | 3 (9.4) | 5 (15.6) | 12 (37.5) | 11 (34.4) |
